# 

**Published:** 1864-08

**Authors:** 


### CHICAGO MEDICAL JOURNAL.

**Terms, $2.00 per Annum.**

### CONTENTS OF THE AUGUST NUMBER.

PAGE.

ORIGINAL COMMUNICATIONS.

Observations on "Congestion." By J. A. A. .......... 337

The Treatment of Healthy Ulcers of the Extremities by "Sealing." By W. B. Slayter, M. D., of Chicago, Graduate Roy. Col. of Physicians of England, etc. .......... 343

Clinical Lectures on Diseases of the Eye. By E. L. Holmes, M. D., of Chicago, Lecturer, on Diseases of the Eye and Ear in Rush Medical College—Chronic Conjunctivitis—Granulated Lids .......... 344

Case of Foreign Body in the Uterus. By John G. Meachem, M. D., of Racine, Wis .......... 350

SELECTED.

On the Progress of Anatomy and Surgery during the present century. By Wm. Fergusson, F. R. C. S., F. R. S., etc .......... 352

Permanganate of Potash in Gonorrhœa. By John G. Rich, M. D., Beachville, Canada West .......... 363

On the Action of the Bromide of Potassium in inducing Sleep. By Henry Behrend, J. R. C. P. E., etc .......... 364

EDITORIAL AND MISCELLANEOUS .......... 367

BOOK NOTICES AND REVIEWS .......... 375

